# The Role of OOH Binding Site and Pt Surface Structure on ORR Activities

**DOI:** 10.1149/2.1071412jes

**Published:** 2014-09-22

**Authors:** Qingying Jia, Keegan Caldwell, Joseph M. Ziegelbauer, Anusorn Kongkanand, Frederick T. Wagner, Sanjeev Mukerjee, David E. Ramaker

**Affiliations:** a Department of Chemistry & Chemical Biology, Northeastern University, Boston, Massachusetts 02115, USA; b Department of Chemistry, George Washington University, Washington, DC 20052, USA; c Electrochemical Energy Research Lab, General Motors Research & Development, Warren, Michigan 48090, USA

## Abstract

We present experimentally observed molecular adsorbate coverages (e.g., O(H), OOH and HOOH) on real operating dealloyed bimetallic PtM_x_ (M = Ni or Co) catalysts under oxygen reduction reaction (ORR) conditions obtained using X-ray absorption near edge spectroscopy (XANES). The results reveal a complex Sabatier catalysis behavior and indicate the active ORR mechanism changes with Pt–O bond weakening from the O_2_ dissociative mechanism, to the peroxyl mechanism, and finally to the hydrogen peroxide mechanism. An important rearrangement of the OOH binding site, an intermediate in the ORR, enables facile H addition to OOH and faster O–O bond breaking on 111 faces at optimal Pt–O bonding strength, such as that occurring in dealloyed PtM core-shell nanoparticles. This rearrangement is identified by previous DFT calculations and confirmed from in situ measured OOH adsorption coverages during the ORR. The importance of surface structural effects and 111 ordered faces is confirmed by the higher specific ORR rates on solid core vs porous multi-core nanoparticles.

In recent years it has been well-established that PtM (M = Co, Ni, Cu) core-shell nanoparticles (NPs) are 5–15 times more reactive for the oxygen reduction reaction (ORR) than pure Pt NPs of comparable size.^1–5^ These core-shell NPs are produced most easily by (electro)chemically dealloying PtM NPs in acid, with the more active M metal leaching out faster allowing formation of the Pt skin. Experimentally and theoretically it has been established that lattice compression of the outer Pt skin covering the PtM rich core is the primary reason for the enhanced ORR activity, as this lattice compression causes a widening and negative shift of the Pt d-band.^1,6^ This weakens the Pt–O bond (the important ORR catalyst’s “descriptor”)^7,8^ and therefore the bonding also of other key intermediates (e.g. OH, OOH, HOOH), because these all involve bonding through the O atom.^9^ Finally the ORR rate has been found to change very uniformly and systematically with M content consistent with Vegards law;^10,11^ the more M atoms in the core the more lattice compression of the skin.^1^

Despite these well-established correlations, it has not been experimentally verified just how the three previously proposed ORR mechanisms change. These mechanisms are differentiated primarily by indicating the molecular species when the di-oxygen bond breaks, namely as O_2_, OOH, or HOOH; the addition of H weakening the di-oxygen bond and therefore making bond breaking easier. How do these rates change with Pt–O bond weakening and hence what intermediates dominate the adsorbate coverage on the Pt surface?

In this work we report direct measurement of the OH, OOH and HOOH intermediate coverage, using in situ X-ray absorption spectroscopy (XAS) during the ORR and show that all three mechanisms play a dominant role at some point with Pt–O bonding weakening (i.e. M/Pt ratio)and that the total yield consists of the sum of all three processes.^12^ We further show that surface effects (Pt structural order) also play a role even on 5–7 nm PtM core-shell NPs. It has been shown previously that on much larger Pt polycrystals surface roughness and order played a role,^13^ and of course the ORR rate has been shown to be significantly different on the 100, 110 and 111 SC planar surfaces.^14,15^ Particles size effects have also been shown to play a role because the fraction of ordered 100 and 111 planes vs. corners and edges change with size,^16–18^ but we use similar sized NPs here, and show that the porosity of the Pt core and skin play a more significant role.

The results are obtained by studying 16 different catalysts, as summarized in Table I, with varying M (M = Co and Ni)/Pt ratio and Pt skin porosity, using XAS data to show how core and skin porosity alter the intermediate OOH coverage and the dominant ORR mechanism. The skin porosity has been changed using alternate gas environments and acids as indicated in Table I to vary the M leaching process rate and thereby the porosity of the NP as discussed elsewhere.^19^

## ORR kinetics and possible intermediate adsorbate coverage

One might ask, what adsorbate intermediates are even expected on the surface during the ORR? Understanding the ORR intermediate species, such as O, OH, OOH, and coverage on catalyst surfaces is critical for understanding the fundamental kinetic mechanisms in a fuel cell.

The ORR kinetics has been well modeled with the rate expression^13,20,21^

[1] $$i=\text{nF}\;k\;c_{O2}\exp(-\alpha \text{FV}/\text{RT})\exp(-\Delta G_{\text{rds}}/\text{RT})\;(1-\theta_{\text{tad}}),$$

where RT/αF is the Tafel slope, and the factors out front are the number, n, of electrons transferred (one) in the rate determining step (RDS), the oxygen concentration, c_O2_, and the exchange rate constant, k, of the rds. Here, V is the overpotential, and the important parameters for this discussion are θ_tad_ and ΔG_rds_. θ_tad_ is the total adsorbate (tad) coverage of all intermediates and other anions and ΔG_rds_ is the activation energy of the rds. The possible intermediates on the surface can be any or all of the following: O, O_2_, OH, OOH, H_2_O_2_, and H_2_O and nearly all of these have been somehow associated with the rds in previous reports far too numerous to review here.

To simplify matters, we follow Koper^8^ who separated the obstacles to an overall reaction into the “kinetic bottleneck” (the rds) vs the “thermodynamic bottleneck”, the latter the primary adsorbate determining step, or more often called^22–24^ the potential determining step (pds). The ORR rds apparently involves getting the initial adsorbate on the surface in either of two mechanisms:

[2] $$\text{Dissociative}:\;\;\;O_{2}+(H^{+}+e^{-})+2^{*}\to {\text{OH}}^{*}+O^{*}$$

[3] $$\text{Non-dissociative}:\;\;\;O_{2}+(H^{+}+e^{-})+{\;}^{*}\to {\text{OOH}}^{*}$$

The fact that eq. 1 models the kinetics, with the proper dependency on c_O2_, pH, n, and V (Tafel slope), points to the reactants on the left side of 2) or 3) being involved in the rds. Further the constant activation energy (ΔG_rds_ = 40 kJ/mol) determined from Arrhenius plots for Pt and Pt-M catalysts,^25^ points to the rds involving perhaps some weakly bonded intermediate, perhaps physisorbed O_2_ or even O_2_ in the inner Helmholtz layer. This is because a more tightly bonded intermediate on the surface, such as OH^*^ or OOH^*^ would change ΔG_rds_ with Pt–O bond strength (i.e. changed Pt surface), assuming a linear Bronsted, Evans, Polanyi (BEP) relation between the activation energy and reactive intermediate free energy, ΔG_ri_; i.e. if ΔG_rds_ = γΔG_ri_ where γ is the BEP constant and ΔG_ri_ should track with the Pt–O binding energy as noted above.^8,26^

The primary adsorbate making up the θ_tad_ will be determined by the pds, which is the most unfavorable step thermodynamically on the surface, or often the last step to become downhill in free energy with increasing potential.^22–24^ That step is the “thermodynamic bottleneck” on the surface and therefore determines the primary adsorbate on the surface and also the magnitude of θ_tad_, not necessarily the rds. This pds has been proposed as one of the reactions:^27–30^

[4] $$\text{Diss}:\;\;\;{\text{OH}}^{*}+(H^{+}+e^{-})\to H_{2}O+{\;}^{*}$$

[5] $$\text{Non-Diss},\;{\text{OOH}}^{*}:\;\;\;{\text{OOH}}^{*}+{\;}^{*}\to O+{\text{OH}}^{*}$$

[6] $$\text{Non-Diss},\;{\text{HOOH}}^{*}:\;\;\;{\text{HOOH}}^{*}+{\;}^{*}\to 2{\text{OH}}^{*};$$

leading to the dissociative, peroxyl, or hydrogen peroxide mechanisms respectively. Here the ^*^ indicates an empty Pt site, and the ad^*^ indicates an adsorbate on a Pt site. In the dissociative mechanism,^27–30^ as the name implies, the Pt–O_2_ bond is so strong that the bonding involves electron transfer from Pt to the O_2_ and therefore simultaneous breaking of the O_2_ bond as in (2). The OOH^*^ and HOOH^*^ mechanisms imply a weaker non-dissociative Pt–O_2_ bond, and therefore 1 or 2 (H^+^+e−) additions are needed to help break the O_2_ bond. Thus the nature of the slow step on the surface, the pds, is very much determined by the nature of the Pt catalysts (particle size, shape and microstructure), because the pds is determined by the strength of the Pt–O bond involving the ORR intermediates adsorbed on the surface (and as noted above also by the potential). The pds primarily determines the ORR rate through the θ_tad_.

## Previous attempts at in situ adsorbate measurements

Unfortunately, obtaining complete clarity on the ORR mechanism requires measurement of the adsorbed reaction intermediates, and this has proven to be very challenging, particularly on real operating electrocatalysts. Experimental verification of the Sabatier principle, i.e. observation of OOH_n_^*^ on the Pt–O weak side and O(H)^*^ on the strong side of the volcano plot, has been elusive (we use OOH_n_ as short hand for OOH or HOOH and O(H) for O or OH).

**T**his work is not the first attempt at obtaining in situ adsorbate coverages during the ORR. We briefly summarize four previously reported in situ adsorbate measurements, which attempted to follow the adsorbates on a Pt cathode in situ with potential.

1. Using ATR-FTIR (attenuated total reflectance-Fourier transform infrared) spectroscopy, Kunimatsu et al.^31^ examined adsorbates during the ORR on a thick Pt polycrystalline film (100 nm particles) in acid. These measurements were preceded by similar measurements in alkaline for a Pt film.^32,33^ Both of these measurements were able to follow an FTIR peak attributed to the O–O stretch of either the superoxide anion, O_2_^−^ or O_2_(ads) on the surface. They proposed an end-on, somewhat tilted orientation of the O_2_^*^, as some component perpendicular to the surface is required in order for it to be IR active. The O_2_^*^ coverage (absent in N_2_ sparged electrolyte) peaked around 0.75 V vs. RHE when moving cathodically, and Kunimatsu et al.^31^ therefore suggested that this was O_2_^*^ preceding the dissociation step, which is in pre-equilibrium with the rds, O^*^ + (H^+^ + e^−^) → OH^*^.
2. Using in situ XANES (x-ray absorption near edge spectroscopy), Erickson et al.^34^ examined the ORR on ~3 nm Pt/C, with high O_2_ flux. The increased XANES near edge intensity or “whiteline” (i.e. ΔW = W(V) − W(0.4 V)) was used to track Pt oxidation. The increased ΔW in the presence of O_2_ vs N_2_ sparged electrolyte was attributed either to increased OH^*^ or O_2_^*^ on the surface, i.e. the ΔW increases for both and thus these could not be distinguished.
3. Using EC-XPS (X-ray photoelectron spectroscopy combined with an electrochemical cell), Watanabe et al.^25^ followed the adsorbates on polycrystalline Pt and PtM (M = Fe, Co, and Ni) bimetallics having a Pt skin. The Pt 4f_7/2_ peak was deconvoluted into contributions attributed to adsorbed H_2_O^*^, OH^*^, and O^*^. They attributed the higher ORR activity of the PtM to the increased O^*^ coverage observed in this case, and therefore indicated the rds is O^*^ + (H^+^+e^−^) → OH^*^.
4. More recently using APXPS (ambient pressure XPS), Casalongue et al.^35^ deconvolved the XPS O 1s peak obtained from an operating fuel cell Pt cathode into 6 different contributions (H_2_O_ml_, H_2_O_ad_, OH–H_2_O, H_2_O–OH, OH–O, OH–O with the underlined atom bound to the Pt) involving H_2_O, hydrated and non-hydrated OH and O with OH. Unfortunately the deconvolution into so many contributing species is not unique as acknowledged by the authors, but they were able to detect the changing coverage of hydrated and nonhydrated OH with potential and O_2_ pressure. These results suggested that under normal hydrated conditions, the pds is OH_ad_ leaving the surface of Pt.

These experimental studies can be summarized as follows: except in the ATR-FTIR experiment, where only O_2_^*^ was followed, it was difficult if not impossible to distinguish mono- from di-oxygen species, OH^*^ from OOH_n_^*^, and in all cases the dissociative mechanism was suggested with O^*^ or OH^*^ as the intermediate in the pds. Thus these experiments never confirmed the Sabatier principle because they could not simultaneously track OOH_n_^*^ vs. OH^*^ or distinguish them clearly from O_2_^*^.

## Experimental

### Catalyst preparation

The dealloyed PtNi_3_/C catalysts were produced via a three step process: 1) the precursor was prepared by an impregnation method, 2) followed by a high temperature annealing step, and 3) chemically dealloyed by acid leaching as described more fully elsewhere.^5^ The final catalyst powder was then manually ground in an agate mortar and pestle prior to preparing the MEA inks.

We report, as summarized in Table I, results for various dealloyed PtM_3_/C (M = Co and Ni) catalysts at 3 different stages of life (after 200 (beginning of life -BOL), 10 k and 30 k (end of life - EOL) cycles giving 16 different PtM catalysts plus pure Pt). These 16 catalysts were demonstrated in fuel cell testing to give excellent oxygen reduction activity and durability, exceeding DOE 2017 targets.^36^ The development of these highly active catalysts and further characterization will be reported elsewhere.^37^ The catalysts are differentiated by the acid used for leaching the M atom (HNO_3_ or H_2_SO_4_ denoted NA or SA) and the use of a post-dealloying thermal anneal in 5% H_2_/N_2_ at 400°C for 4 hrs (denoted un-annealed or annealed). ORR specific activities (mA/cm^2^, at 900 mV in 50 cm^2^ MEAs with 0.1 mg/cm^2^ Pt loading^36^ were measured for each catalyst. They were characterized structurally by HAADF ((high-angular annular dark field))/EELS (electron energy loss spectroscopy),^37^ Pt L_3_ and Ni K-edge EXAFS, Pt L_3_ edge XANES (provided in Supplemental Figure S1–8; see the Supplemental Material link in the online version of this article), as well as Ni K Δμ XANES with full structural results to be reported elsewhere.^38^ In general the particles are well formed 5–7 nm single core-shell particles (i.e. with a PtNi_x_ core and a ~0.6 nm thick Pt shell).

### MEA tests

The MEAs were fabricated by a standard catalyst-coated membrane (CCM) method via draw down with a Meyer rod and subsequent decal transfer. This information is described more fully elsewhere.^39^ The above-prepared 50 cm^2^ MEAs were tested for their cathode ORR kinetic activities under H_2_/O_2_ (anode/cathode); H_2_/O_2_ stoichiometries of 2.0 and 9.5, 100% relative humidity, cell temperature of 80°C, and at a back pressure of 150 kPa_abs_. In voltage cycling tests, 200 sccm H_2_ into anode and 50 sccm N_2_ into cathode were admitted at 150 kPa_abs_. The cell voltage was swept at 50 mV · s^−1^ between 0.6 and 1.0 V (RHE) in a triangular profile for up to 30,000 cycles. The MEAs were subjected to the cathode catalytic activity (H_2_/O_2_) and the H_2_/air performance tests after 200, 10,000, and 30,000 voltage cycles. The data collection procedures and conditions, specifically the ORR and ECSA measurements,^40^ were performed according to the guidelines set by the USCAR Fuel Cell Tech Team (http://www.uscar.org/guest/view_team.php?teams_id=17). The mass and specific activities are summarized in Ref. 40 (page 9). A symbol translation table is provided in Supplemental Table S1 as the catalyst symbols adopted in Ref. 40 (page 7) are different from those used here (Table I).

### Electrode preparation and XAFS data collection

The electrode inks for the EXAFS electrodes were composed of 1:1 (wt%) 18.2 MΩ purity deionized water (Millipore) and 2-propanol (HPLC-grade, Aldrich), a 5 wt% Nafion solution (Aldrich), and the catalyst powder. The composition was chosen to give a final electrode with a dry Nafion loading of 5 wt%. The ink was hand-painted onto a Zoltek carbon cloth and dried for 15 minutes in a 65°C vacuum oven between coats. The final Pt and Ni geometric loadings were chosen to give ~0.05 edge heights at the Pt L_3_ and Ni K edges, respectively. All data were collected in the fluorescence mode at beamline X3B at the National Synchrotron Light Source, Brookhaven National Lab. All of the experimental data were collected in conjunction with the appropriate reference foils to aid in energy alignment and normalization. A flow-through cell with continuously pumped 0.1M HClO_4_ (GFS Chemicals) was constantly sparged with either argon (nitrogen) or oxygen gases (both high-purity). A high-purity, multiply-wound Au wire (+99.95%, Alfa Aesar) was used as the counter electrode, and a sealed saturated Ag/AgCl electrode (measured −0.283 V vs. reversible hydrogen electrode, RHE) served as the reference electrode. Potentiostatic control was maintained with an Autolab PGSTAT302N potentiostat/galvanostat (MetroOhm/Brinkmann). The voltage cycling limits were 0.05 to 1.1 V vs. RHE. Data collection was performed at the chosen potentials held during anodic sweeps. Before each measurement, the cell was held for 5 minutes to reach a pseudo-steady-state, which is justified by the stable current density and the reproducibility of the data. The electrode was fully cycled following each potential hold in order to clean the electrocatalyst surfaces after each potential hold.

## Results and Discussion

### Catalysts structure and reactivity

**Figure 1:**
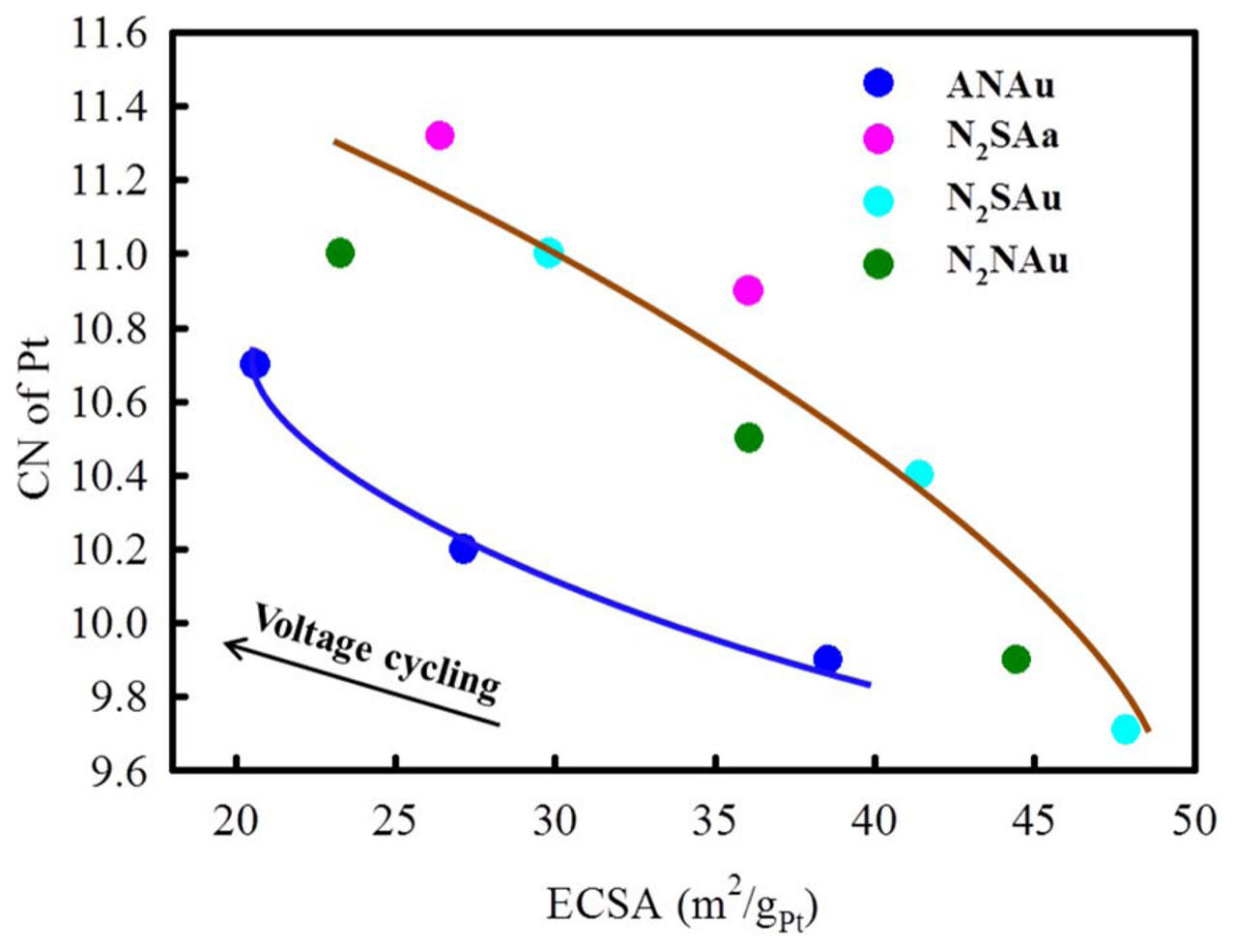
Pt-Pt coordination numbers obtained from in situ Pt L_3_ EXAFS data as a function of H_upd_ measured electrochemically active surface area (ECSA)^40^ for the four catalysts indicated. Separate curves are drawn through the points for those catalysts prepared in an air (A) environment (blue) vs. those in an N_2_ environment (red) distinguishing the more porous cores (ANAu) from the other more solid cores.

Previously reported HAADF imaging data^40^ for the as-prepared NAu (nitric acid un-annealed) catalysts dealloyed in an air vs. N_2_ environment (i.e. resulting in ANAu vs N_2_NAu dealloyed catalysts in Table I), showed the multi-core and porous structure of the ANAu catalysts vs. the more solid core in the N_2_NAu case. The average NP size is around 5–7 nm, with the ANAu NPs having slightly larger size and greater size distribution. Consistent with this, Fig. 1 showing the total coordination numbers (CN) of Pt, obtained from EXAFS analysis (provided in Supplemental Table S2), reveals that this difference in porosity remains even after 30 k electrochemical cycles. This cycling, done according to DOE (Department of Energy) standards^41^ is utilized to perform an accelerated stress test. The H_upd_ measured electrochemically active surface area (ESCA), as we move from the BOL (beginning of life), to 10 k and 30 k cycles (right to left) decreases because of growth in particle size and perhaps some removal of the under-coordinated sites at the particle corners and edges with cycling. But most importantly, the ANAu catalysts show a significantly lower CN at the same ECSA. This of course arises because of the multicore porous nature of the ANAu catalysts leaving Pt atoms uncoordinated at the edges/corners of the pores, throughout the cycling process. In contrast, the M-edge XAS data^38^ utilizing Δμ analysis enables an in situ determination of when O atoms electrochemically adsorbed on the Pt surface penetrate the Pt skin to gain access to the underlying M atoms. These data show that the penetration potential of the different catalysts all merge to the same value after 30 k cycles and are close already at 10 k cycles. These results suggest that the thickness/robustness of the Pt skins are similar after extensive cycling, but that the porosity existing in the core remains and this subtly alters the order existing in the Pt skin that affects the ORR.

**Figure 2:**
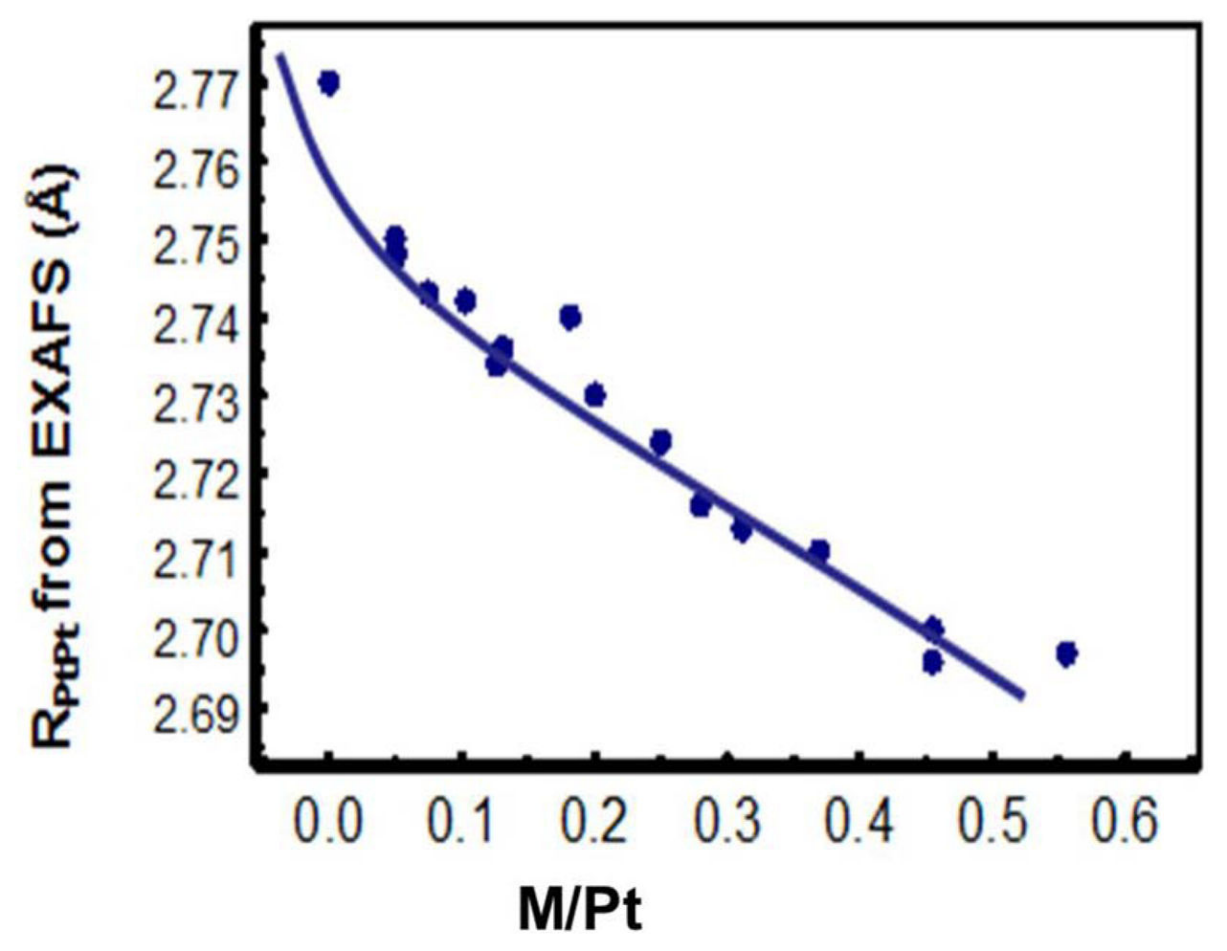
Plot of Pt–Pt bond distance, R_PtPt_ for 16 PtM catalysts and for Pt as obtained from EXAFS analysis vs. the M/Pt ratio obtained from Energy Dispersive X-ray Spectroscopy (EDS) analysis after dealloying and cycling.

**Figure 3:**
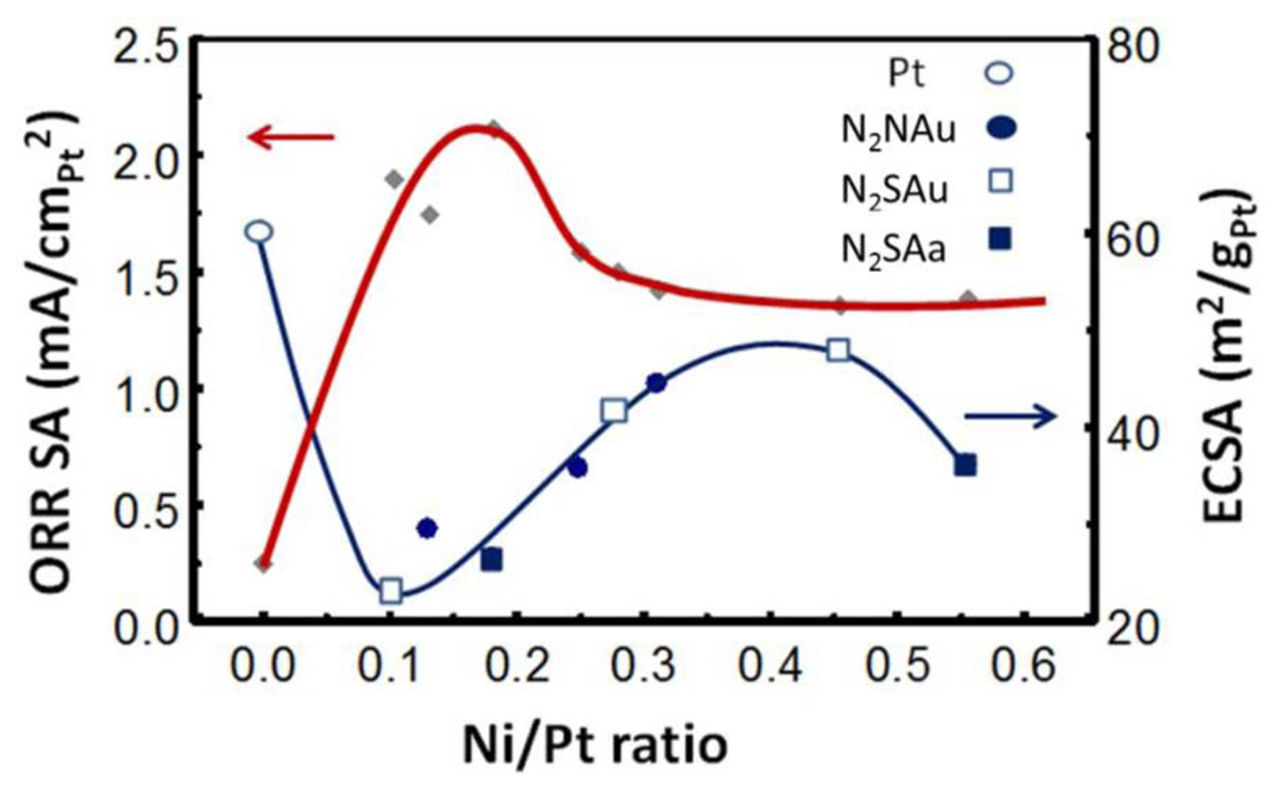
ORR specific activity along with the electrochemical active surface area (ECSA)as measured with H_upd_. The points on the ECSA curve indicate the catalysts with notation in the legend as in Table I. The BOL catalysts have the largest Ni/Pt ratio in each case, and ECSA decreases with cycling due to particle growth. Only BOL and 30 K results are available for the N_2_SAa cataysts.

Figure 2 shows a plot of Pt-Pt bond distance for the 16 catalysts as obtained from EXAFS analysis vs. the Ni/Pt ratio obtained from Energy Dispersive X-ray Spectroscopy (EDS) analysis after dealloying and cycling. The nearly linear slope confirms Vegard’s law, and shows the nearly linear compression of the Pt-Pt distance with Ni/Pt ratio. The EXAFS data gives the average Pt-Pt distance throughout the entire cluster (core + skin), but the uniformly varying data in Fig. 3 strongly indicate that this general bulk compression also determines the strength of the Pt–O bond at the outer Pt skin. This is indicated by the systematic variation vs Ni/Pt ratio of the ORR specific activities (SA) in Fig. 3.

### Δμ XANES results

Δμ XANES data on the dealloyed NPs enable a direct measure of the OH^*^, OOH^*^ and HOOH^*^ adsorbate intermediates on the Pt. The Δμ XANES data defined as, Δμ = μ (A/Pt) − μ(Pt) is surface sensitive because it involves the difference between the XAS absorption, when adsorbates are present, μ(A/Pt), vs when they are nearly absent μ(Pt), thus highlighting the small changes occurring due the adsorption. Small NPs are required for this measurement, as the ratio of surface atoms to all atoms are nearly negligible for the large polycrystals used by Stamenkovic et al.^13^ Theoretical multiple scattering FEFF8^42^ calculations enable identification of the different Δμ signatures for each adsorbate as reported previously in the literature.^12,43–50^

FEFF8 calculations on small Pt_6_ model clusters to obtain theoretical signatures, Δμ = μ(Ad/Pt_6_) − μ(Pt_6_), have been shown to produce reliable Δμ signatures in excellent agreement with experiment for O, OH, CO (bridged and atop), H (3-fold vs atop), and other small adsorbates.^12,43–48^ A Pt_6_ cluster has been used often to model the experimental Pt clusters, as this highly asymmetrical cluster provides for all of the possible common binding sites^51^ (fcc, hcp, bridged, and atop) and is asymmetrical so that it does not introduce any “surface resonances”, which might arise from a more symmetric cluster,^52^ and yet the cluster is sufficiently large to account for the change in Pt-Pt scattering and new Pt-Ad scattering introduced by the adsorbate. Recently, calculations with increasingly bigger clusters (Pt_6_, Pt_13_ and Pt_25_) were compared and found to give similar Δμ signatures,^53^ and DFT calculations on slabs were shown to also give similar signatures as FEFF8 for O/Pt and CO/Pt.^54,55^ These calculations show that the dominant contributions to Δμ are “local” to the Pt–O bond, and hence small clusters already give qualitative agreement with experiment, certainly sufficient to identify the binding site. Since the experimental data is an average over a wide array of particles sizes and shapes in a catalyst, any further attempt to improve on the model cluster to find more quantitative agreement between the experimental and theoretical signatures is deemed to be fruitless.

**Figure 4:**
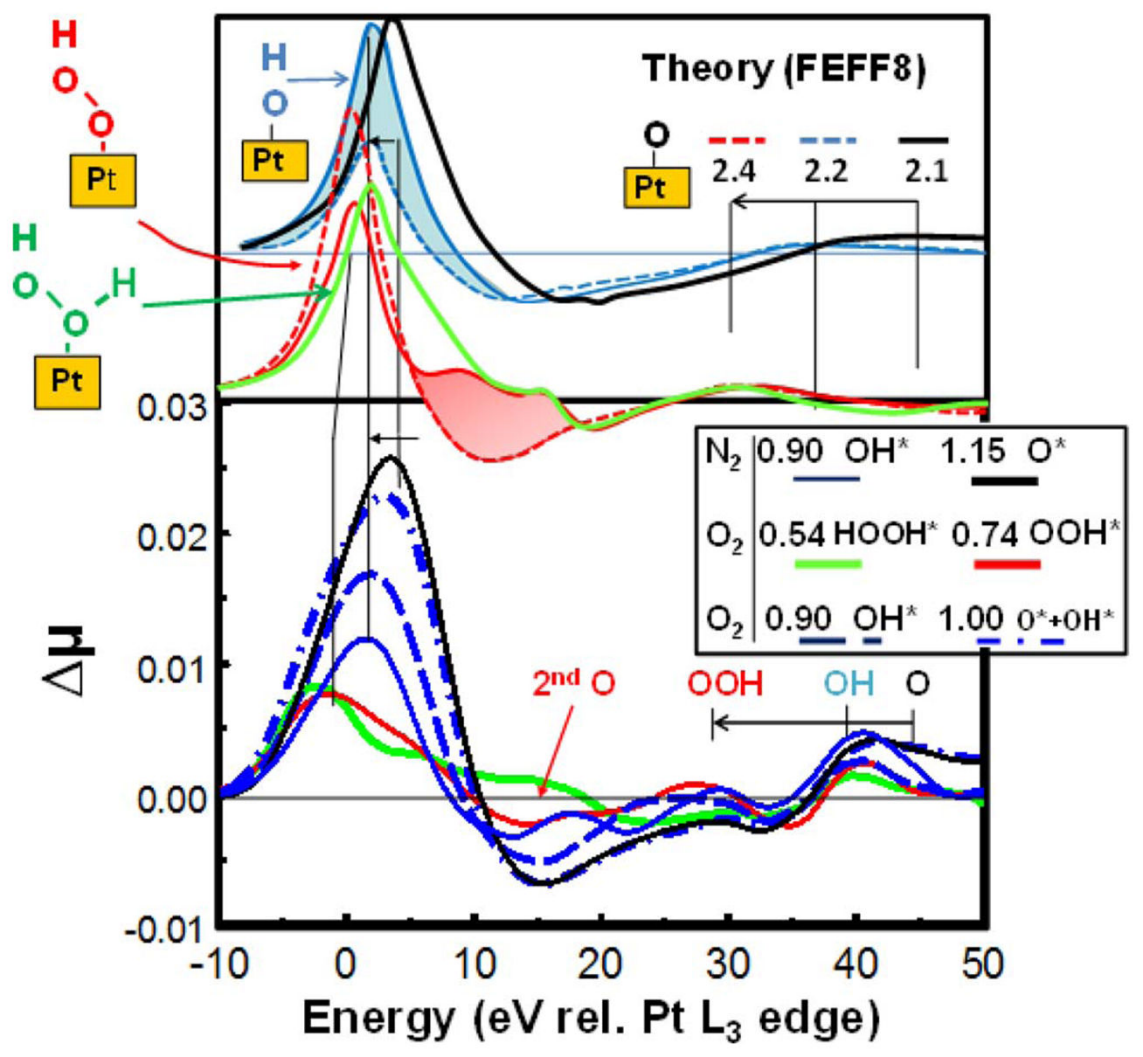
Δμ = μ(V, O_2_ or N_2_ sparged) − μ(0.54, N_2_ sparged) for the N_2_SAu catalyst after initial break-in (BOL) at the indicated potentials. Also indicated are FEFF8 results for the indicated adsorbates with Pt–O bond lengths as indicated in Å producing the shifts of the 30–45 eV peak (dashed lines show Δμ for just the O atom at the appropriate Pt–O bond length, solid for the full adsorbate such as OH^*^ in blue and OOH^*^ in red). The contribution from the second O in the OOH^*^ is highlighted in red, that from the H in the OH in blue, as determined from the difference in Δμ with and without the second atom.

Pt L_3_ XAS data were obtained in situ with the catalysts sparged in either N_2_ or O_2_. In N_2_ any adsorbates must come from water activation, which generally occurs well above 0.54 V (rel. RHE), thus μ(0.54 N_2_) is taken as the reference as in our previous work. ^43–48^ Fig. 4 gives Δμ = μ(V,O_2_ or N_2_)−μ(0.54,N_2_) for catalysts at several potentials showing how the Δμ signatures change with potential. Fig. 4 also gives FEEF8 signatures for O^*^, OH^*^, OOH^*^, and HOOH^*^ with adsorbate orientations as shown, and binding in the atop Pt sites. The position of the peak around 30–45 eV directly reflects the Pt–O bond distance reminiscent of EXAFS, and these are indicated in the figure going as O < OH < OOH; i.e. consistent with O bond order conservation. The feature around 5–20 eV reflects the x atom bonded on the outer side of the O atom in Pt–O-x, either nothing, O, or O + H. The second O backscatters the photoelectron so it produces the feature at 10–20 eV, and the H alters the feature between 3–10 eV as shown by the shaded regions. Comparison with the experimental signatures strongly suggest that with N_2_ sparging at 0.9 V we have OH^*^ and at 1.15 V mostly O^*^ as shown many times before.^46–48^ With O_2_ sparging, at 0.54 V better agreement is evident with HOOH^*^, at 0.74 V better with OOH^*^, and at 0.9 with OH^*^ and at 1.0 V apparently reflecting O^*^ + OH^*^.

The results above confirm a di-oxygen species on the surface orientated with axis oblique to the Pt surface in an atop site with O_2_ sparging. This is consistent with theoretical calculations.^56^ DFT calculations on a OOH/Pt_3_ cluster indicate that OOH^*^ prefers a Pt atop site with axis oblique to the surface,^57^ with Pt–O = 2.0 Å and O–O = 1.4 Å^58^ in reasonable agreement with that found by comparison of FEFF8 and experimental signatures, 2.4 and 1.4 when OOH is bound to a 3–5 nm Pt particle. Similar calculations suggest HOOH^*^ would orient as illustrated.^56,58^ Finally calculations indicate a weakly bonded O_2_^*^ (0.3–0.5 eV compared to O^*^ at 3.3–3.7 eV) would bond in a bridged site or in an fcc hollow site (atop-hollow-bridge); i.e., nearly parallel not oblique to the surface,^59^ and therefore would give a Δμ significantly different from OOH_n_.

**Figure 5:**
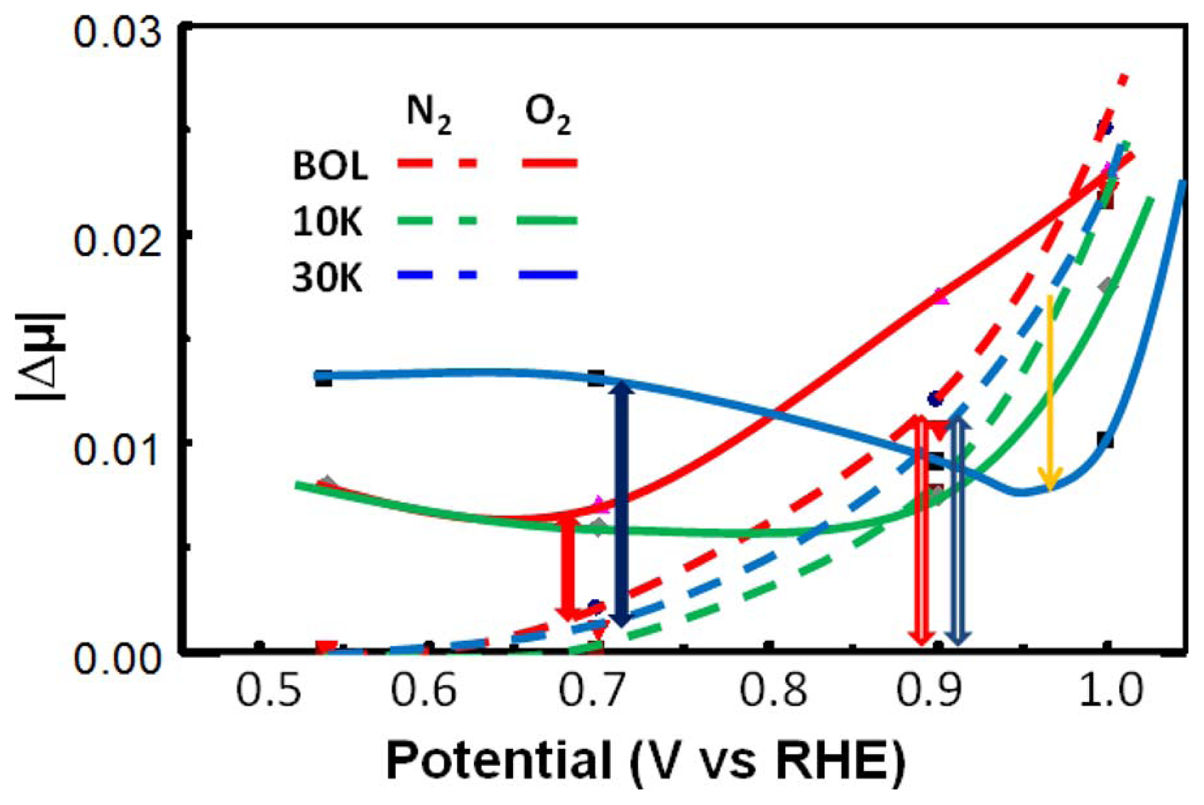
Plot of |Δμ| magnitudes obtained from the peak heights around 0–5 eV above the Pt L_3_ edge for the N_2_SAu catalyst at 3 stages of life, BOL (200 cycles) and after 10 k and 30 k cycles(EOL), performed with DOE protocol, with N_2_ or O_2_–sparged electrolyte as indicated. The filled block arrows around 0.7 V indicate the relative coverage of OOH^*^ at 700 mV (i.e. OOH_n_^*^ in the presence of O_2_) and the open block arrows the coverage of OH^*^ in N_2_ (i.e. OH^*^) near 900 mV. The yellow arrow indicates the drop in OH^*^ around 0.95 V (vs. RHE) due to competition with O_2_^*^ for Pt sites.

Fig. 5 shows the amplitude of the Δμ maximum around 0–5 eV-above the Pt L_3_ edge for the N_2_SAu catalysts, as shown in Fig. 4 as a function of potential after either N_2_ or O_2_ sparging of the HClO_4_ electrolyte at the 3 different stages of life (BOL, and after 10 and 30 k cycles). Note that in N_2_ sparged electrolyte, the OOH_n_^*^ coverage is negligible as the OH^*^ comes just from water activation. In O_2_ sparged electrolyte, the dioxygen species compete with the OH^*^ species, so (as highlighted by the yellow arrow), the OH^*^ coverage is decreased compared to the N_2_ sparged electrolyte. The relative OH^*^ at 0.9 V and OOH_n_^*^ coverage at 0.7 V given in Fig. 5 are those indicated by the height of the arrows for BOL and 30 K, and similar results for the 10 K data. The different potentials (0.7 in O_2_ or 0.9 V in N_2_) for the OOH_n_^*^ and OH^*^ coverages, respectively, allow for the best separation of adsorbate species, when one or the other dominates.

The significant adsorbed dioxygen species evident down to 0.54 V in Fig. 5 confirms the effectiveness of our flow through cell, which pumps O_2_ saturated HClO_4_ electrolyte constantly to the cathode. It has been shown previously,^12^ using Δμ XANES in a full operating fuel cell, that at the O_2_ diffusion limit the coverage of nearly all adsorbed dioxygen species goes to zero as expected. In the fuel cell this occurred at high current and low cell potential, around 0.2–0.4 V. Similarly, Erickson et al,^34^ using a specially designed high oxygen flux cell containing a poly(dimethylsiloxane) membrane with high permeability toward oxygen, found that the diffusion limit fell below 0.2 V. This is in contrast to typical RDE plots which typically show the diffusion limit occurring already around 0.85 V because of the low solubility of O_2_ in water.^60^ The much lower potential at the diffusion limit in fuel cells occurs in part due to the much larger IR losses at high currents in fuel cells,^12^ but mostly due to the higher O_2_ concentrations occurring in a fuel cell. In an *in operando* PEM fuel cell, humidified O_2_ is continually pumped to the cathode via the gas diffusion layer. The continuous flow through cell utilized in this work is apparently also providing sufficient O_2_ concentrations to the cathode to well below 0.5 V.

**Figure 6:**
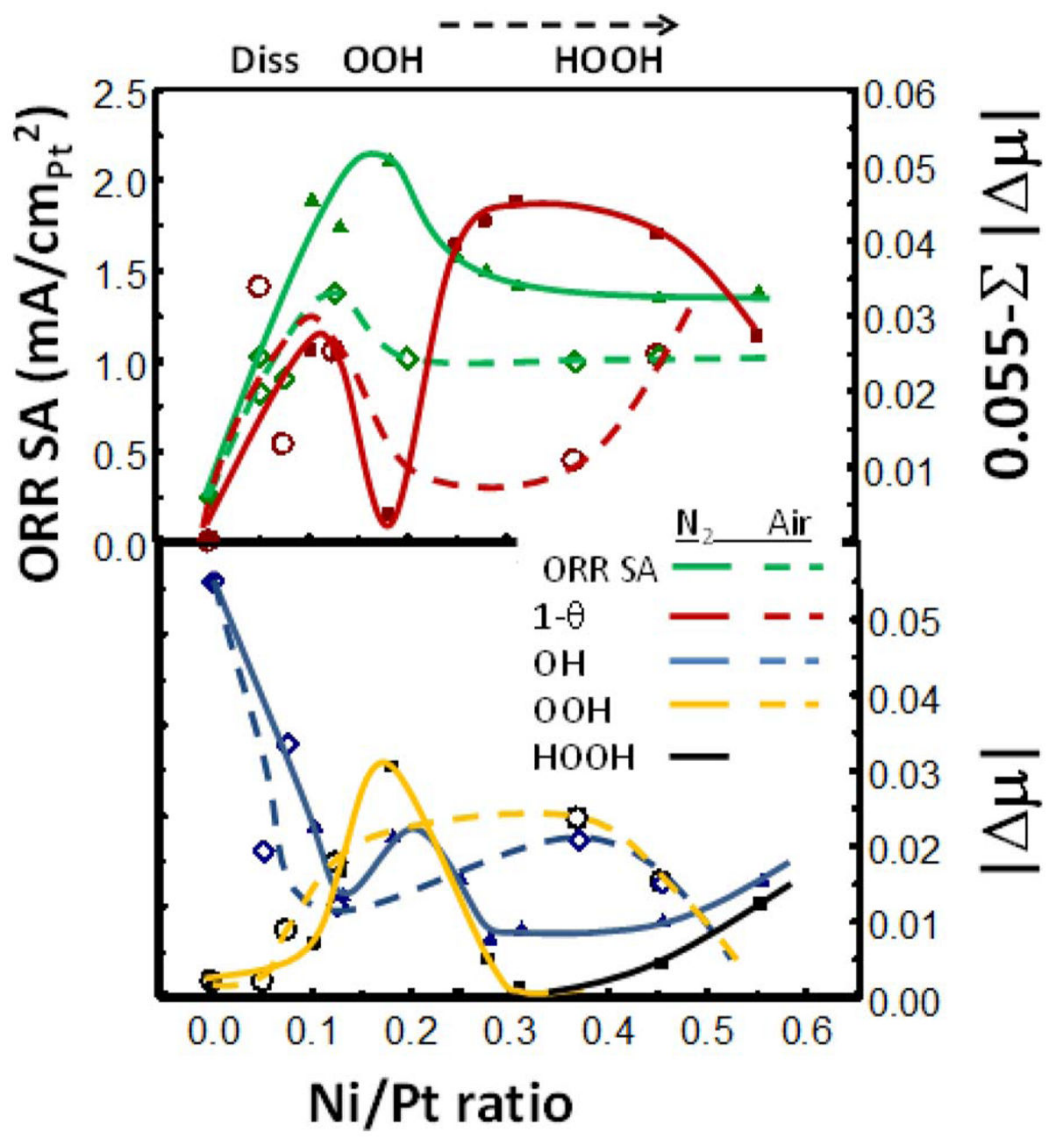
Comparison of data for those catalysts de-alloyed in N_2_ (solid) and those in air (porous). Top: Plot of ORR specific activity (SA) and 0.055 – Δμ_OH_–Δμ_OOH_ – Δμ_HOOH_ representing the number of empty Pt sites, (1−θ_tad_), during the ORR as a function of the Ni/Pt ratio. Bottom: Plot of Δμ_OH_ measured in N_2_ sparged electrolyte at 0.9 V RHE and Δμ_OOH_ measured in O_2_ sparged electrolyte during the ORR.

Figure 6 collects the Δμ and reactivity data from all 16 catalysts and shows how the OH^*^ and OOH^*^ coverages change with Ni/Pt near 0.8 V. It also shows that the specific area (SA) ORR activity of the solid NPs is indeed about 1.5–1.8 higher than for the porous NPs. The SA peaks with a typical asymmetric volcano curve at a Ni/Pt ratio of 0.17 (a bit higher for the solid case) and above 0.27 the additional Ni appears to have little effect. These SA ORR activity curves can be regarded as the NP equivalent of the previous polycrystalline data reported by Stamenkovic et al.^13^ showing the difference between leached PtM (Pt skeleton) and annealed PtM (Pt skin) with M consisting of Ti, V, Fe, Co, and Ni. Similar to Fig. 6, their data showed increased SA for the annealed skin, with the volcano maximum moving to larger d-band shift (decreased Pt–O bond strength). Collectively these data are all consistent, and suggest strongly that the 111 planes are uniquely active compared to the other more open planes and corners/edges. Indeed these authors suggest that the most active Pt_3_Ni (111) SC catalysts known to exists, falls qualitatively on these volcano curves, and is the most active because of the SC 111 planes.^13^ What is it about the Pt atoms on the 111 planes that make them the most active?

The results below Ni/Pt = 0.2 show typical Sabatier catalytic behavior. On the strong Pt–O bonding side of the volcano (low Ni/Pt ratio), relatively fast dissociative O_2_ adsorption occurs with the slower step being H addition to OH^*^ enabling it to leave the surface. As the Pt–O bond weakens with increasing Ni/Pt, the OH^*^ coverage decreases, and the OOH^*^ coverage increases due to the increasing difficulty of breaking the di-oxygen bond. The cross-over in coverage occurs right at the volcano maximum. Eq. 1 showed the simple rate expression,^13,20,21^ i ∝ exp(−ΔG_act_/RT) (1−θ_tad_). We show in Fig. 6 a quantity equal to 0.55–μ_OH_–μ_OOH_–μ_HOOH_ representing the relative 1−θ_tad_, and it peaks very near the volcano maximum for the porous NPs as expected for typical Sabatier bahvior. The ORR rate does not fall dramatically beyond Ni/Pt = 0.2 however because now the OOH mechanism takes over going as exp(−ΔG_act_/RT) θ_OOH_. The difference in rate dependece (1−θ_tad_) vs θ_OOH_ for these two cases occurs because with the dissociative O_2_ mechanism, a later H addition to OH^*^ cleans off the surface and is the potential determining step (pds), but with the OOH mechanism the first H adddition to O_2_ is the pds (i.e. now the pds and rds are the same or the pds even precedes the rds).^8^

A significant difference occurs for the solid NPs, indeed 1−θ shows a double peak behavior with a minimum at the SA volcano peak. This 1−θ minimum falls right where the OOH^*^ coverage shows a narrow maximum, much narrower than for the porous NP case. This narrowing is believed to arise because on the solid NPs H addition to OOH^*^ is apparently able to occur at a much faster rate, changing OOH^*^ to HOOH^*^, which is then able to dissociate the di-oxygen bond much more easily. As a result OOH^*^ coverage decreases and some HOOH^*^ begins to build up only after the Ni/Pt ratio goes above 0.35. Thus each of the 3 di-oxygen dissociation mechanisms dominates at some point in the case of the solid NPs. The rate going as exp(−ΔG_act_/RT) (1−θ_tad_) now gives two separated peaks, the first as the dissociative O_2_ mechanism switches to OOH and the second as OOH switches to HOOH. Between these two peaks the rate goes as exp(−ΔG_act_/RT)θ_OOH_, when the OOH mechanism dominates. Summed together only one assymetric and broader ORR volcano is visible.

**Figure 7:**
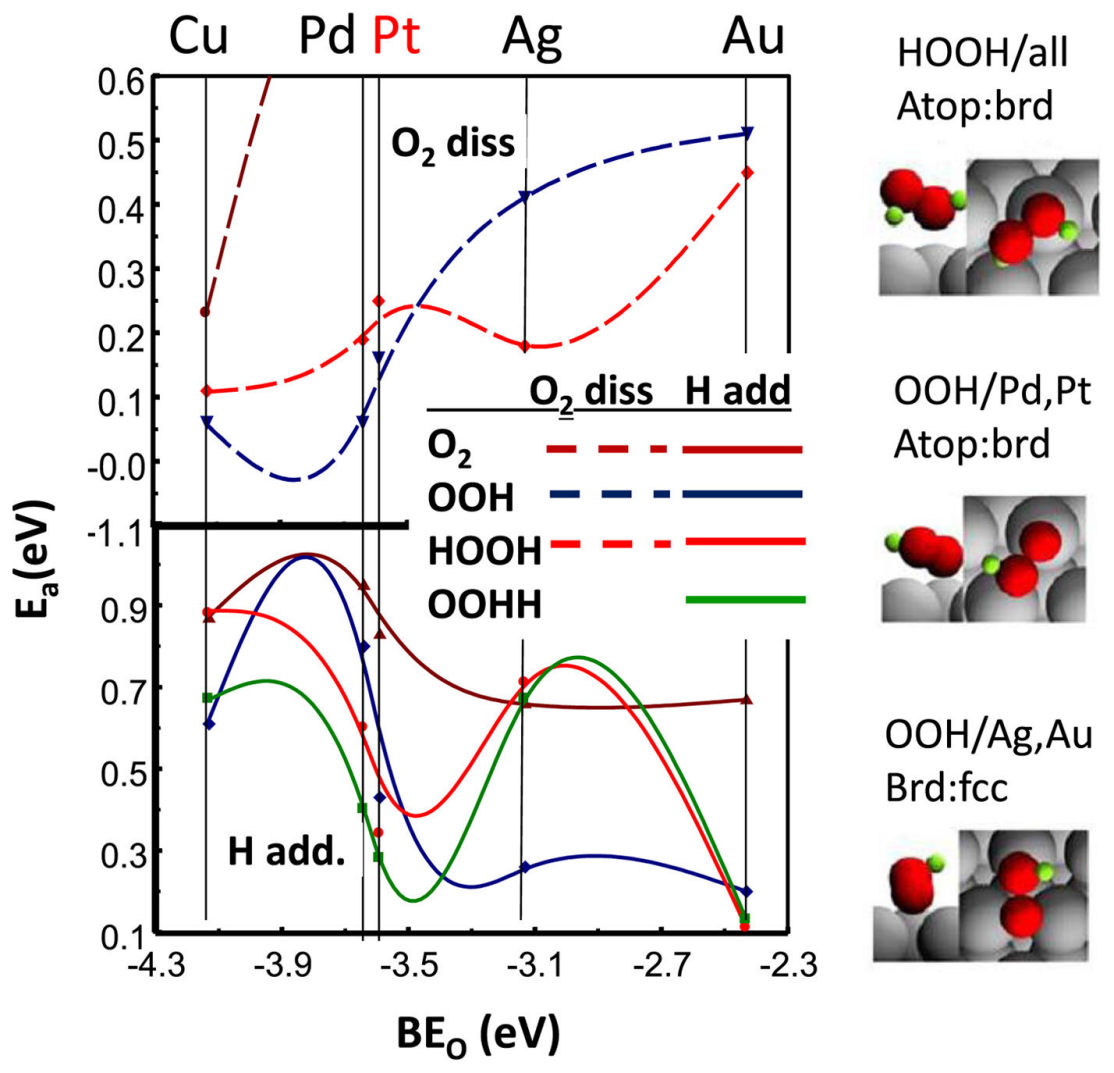
Activation energies (E_a_) obtained from DFT slab calculations for H addition (bottom) and O–O dissociation (top) as reported by Ford et al.^61^ A least squares interpolation of the E_a_’s is indicated between the M atoms. Schematic illustrations of the optimal binding sites of OOH and HOOH on Pt and Ag as estimated from the DFT calculations are indicated at the right. Adapted from Ref. 61 with permission from Elsevier Publishing, 2014.

The in situ Δμ XANES results suggest that the OOH^*^ coverage markedly decreases in magnitude on 111 planar sites, because of a much faster addition of H to form HOOH^*^ that can then undergo faster dissociation due to the weaker di-oxygen bond. Therefore the critical aspect must be something involving the binding of OOH^*^ on these sites; i.e. a surface structural effect involving the OOH^*^ bonding on the 111 sites must occur, which does not occur on the edge/corners or 100 sites. Recent DFT calculations reported by the Mavrikakis group^61^ for ORR on Cu, Pd, Pt, Ag, and Au (111) planes, and reasonable least squares interpolation in between, reveals this significant change in OOH^*^ binding upon moving from Pt to Ag, as highlighted in Fig. 7. This rearrangement dramatically decreases the activation energy for H addition to OOH^*^ producing a minimum between Pt and Ag, i.e. right where PtM NPs would fall. Fig. 7 shows that the most stable binding of OOH^*^ shifts from a atop/bridged site (closest O in the atop site, OOH over a bridge site) on Pt, to a bridge/fcc site (closest O in a bridged site, OOH over the fcc site) on Ag, Au. In contrast, the most stable site for the HOOH^*^ is the same on all the M (111) surfaces. Therefore H addition to OOH^*^ proceeds relatively easily over Pt that does not involve a rearrangement, but requires a much higher activation energy over Ag because of the required rearrangement. The minimum falls in between (i.e. for PtNi) when the OOH^*^ binds the weakest, but has not yet changed binding sites requiring the rearrangement. This explains the trend in enhancement factors reported by Wagner et al.^62^ The enhancement factor of 111 sites over other facet sites (111/100 or 111/110) is .82 and 0.35 respectively for Pt, but 20 and 8.2 respectively for PtNi. The enhancement factor is larger for PtNi when the Pt–O binding is near the minimum in Fig. 7.

Figure 7 also reveals some other basic points. Note the general increase in activation energy, E_a_, for bond breaking and decrease of E_a_ for H addition with Pt–O binding energy (BE_o_), but as the results show, this trend is not monotonic but contains the minimum because of the OOH^*^ site rearrangement. Note also the general decrease in E_a_ for breaking the O bond as the number of H atoms increase, the addition of H weakening the O–O bond. Results for H addition to the aquioxyl species (HHOO^*^) is also shown because this path is feasible, and HHOO has a very low E_a_ for bond breaking.^61^

In summary, lattice compression (d-band shift) weakens the Pt–O bonding on NPs causing the OOH mechanism to dominate the ORR beyond Ni/Pt = 0.2. The OOH^*^ site binding rearrangement on the 111 faces falling at Pt–O bond strength between Pt and Ag; namely around PtM, provides for optimal facile H addition enabling faster ORR reduction. Solid NPs have a larger number of these 111 planar sites because Pt atoms near the pores in the porous NP’s contain more non-coordinated edge sites. Perhaps even more important, if we think of the porous NPs consisting of a Pt “blanket” over multicores, then the compression effect on the Pt skin might be very uneven, and this would also reduce the extent of order or number of 111 planes significantly; i.e. the Pt skin or blanket may have “wrinkles” and defects because of the underlying porous core structure. In either case, it is clear that significant surface effects exists particularly for these dealloyed PtM core-shell NPs, because of the important OOH^*^ lattice rearrangement occurring right at the optimal Pt–O bond strength.

## Supplementary Material

sopporting materials

## Figures and Tables

**Table I:** Summary of catalysts studied.

Catalyst symbol	Pre-cursor	Dealloying gaseous environment	De-alloying acid	Post annealing^1^	Life stages measured^2^	Morphology^3^
APtCo	PtCo	Air	HNO_3_	No	BOL	SC-NS
APtCo_3_	PtCo_3_	Air	HNO_3_	No	BOL,10 k,30 k	50% MC-PS,50% SC-NS
ANAu	PtNi_3_	Air	HNO_3_	No	BOL,10 k,30 k	MC-PS
N_2_NAu	PtNi_3_	N_2_	HNO_3_	No	BOL,10 k,30 k	SC-NS
N_2_SAu	PtNi_3_	N_2_	H_2_SO_4_	No	BOL,10 k,30 k	SC-NS
N_2_SAa	PtNi_3_	N_2_	H_2_SO_4_	Yes	BOL,10 k,30 k	SC-NS

1 Post annealed with a thermal treatment in 5% H_2_/N_2_ at 400°C for 4 hrs.

2 XAS data at beginning of life; i.e. after around 200 cycles (BOL) or after 10,000 or 30,000 cycles (EOL) following DOE protocol.

3 Morphology description obtained from TEM data: either multi- or single core PtM_x_ (MC or SC) with porous or non-porous Pt skin (PS or NS).^40^
