# Supplementary material for: The Role of OOH Binding Site and Pt Surface Structure on ORR Activities
Source: J Electrochem Soc. Author manuscript; Available in PMC 2015 Sep 22. (PMC4501392; doi:10.1149/2.1071412jes)
Supplement: sopporting materials [file NIHMS702725-supplement-sopporting_materials.pdf]

SUPPLEMENTARY INFORMATION

The role of OOH binding site and Pt surface structure on ORR activities

Qingying Jia[†], Keegan Caldwell[‡], Joseph M. Ziegelbauer[§], Sanjeev Mukerjee[†] and David E. Ramaker[‡]

[†] Dept. of Chemistry & Chemical Biology, Northeastern University, Boston, MA 02115

[‡] Department of Chemistry, George Washington University, Washington, D.C. 20052

[§] Electrochemical Energy Research Lab, General Motors Research & Development, Warren, MI 48090

Table S1: Symbol translation table

Catalyst symbol used here	Catalyst symbol used in Ref. 40 (Page 7)	Pre-cursor	Dealloying gaseous environment	De-alloying acid	Post anneal-ing[2]
ANAu	176NA	$PtNi_3$	Air	HNO_3	No
N_2NAu	280NA	$PtNi_3$	N_2	HNO_3	No
N_2SAu	280SA	$PtNi_3$	N_2	H_2SO_4	No
N_2SAa	280SA-AN	$PtNi_3$	N_2	H_2SO_4	Yes

EXAFS analysis

Table S2: Summary of EXAFS results*

Catalyst symbol	Cycling stage	R_{Pt-Pt}	R_{Pt-Ni}	R_{Ni-Ni}	N_{Pt-Pt}	N_{Pt-Ni}	N_{Ni-Pt}	N_{Ni-Ni}	σ^2_{Pt-Pt}	σ^2_{Pt-Ni}	σ^2_{Ni-Ni}	R-factor
		($\Delta R = \pm 0.008$Å)[a]			($\Delta N = \pm 0.9$)[b]		($\Delta N = \pm 0.5$)[b]		($\Delta\sigma^2 = \pm 0.007$)[c]			
ANAu	BOL	2.738	2.630	2.572	9.2	0.7	7.5	4.4				0.005
	10 k	2.743	2.650	2.575	9.6	0.6	6.8	4.1	0.005	0.010	0.009	0.007
	30 k	2.748	2.650	2.577	10.3	0.4	5.9	4.7				0.004
N_2NAu	BOL	2.713	2.611	2.570	8.1	1.8	6.9	5.5				0.007
	10 k	2.724	2.631	2.574	9.1	1.2	6.7	5.5	0.004	0.010	0.011	0.009
	30 k	2.742	2.631	2.543	10.6	0.4	6.3	5.6				0.009
N_2SAu	BOL	2.696	2.601	2.581	7.3	2.5	4.8	7.7				0.010
	10 k	2.716	2.604	2.574	8.2	2.2	3.8	7.4	0.007	0.005	0.011	0.012
	30 k	2.736	2.600	2.580	9.7	1.3	3.7	7.4				0.008
N_2SAa	BOL	2.693	2.598	2.578	8.8	2.1	5.2	7.1	0.007	0.005	0.011	0.006
	10 k	2.717	2.600	2.575								
	30 k	2.734	2.604	2.570	10.4	0.9	4.6	6.9	0.007	0.005	0.011	0.010

*All the data were collected at 0.54 V vs RHE (double layer region) in N_2-saturated 0.1 M $HClO_4$ electrolyte except for the Pt L_3 edge data of the 10k-cycled N_2SAa catalyst that were collected under *ex situ* conditions (thus only the bond distance values are given). S_0^2 fixed at 0.766 and 0.682 for Pt and Ni, respectively as obtained by fitting the reference foils. Fits were done in *R*-space, $k^{1,2,3}$ weighting at Pt L_3 and Ni k edges simultaneously. For Pt, 1.1< R < 3.4 Å and Δk = 2.95 - 12.14 Å^{-1} were used; for Ni, 1.2 < R < 3.0 Å and Δk = 1.87 – 11.93 Å^{-1} were used. [a,b,c]Values represent the largest statistical errors of all of the least-squares fits determined by ARTEMIS.

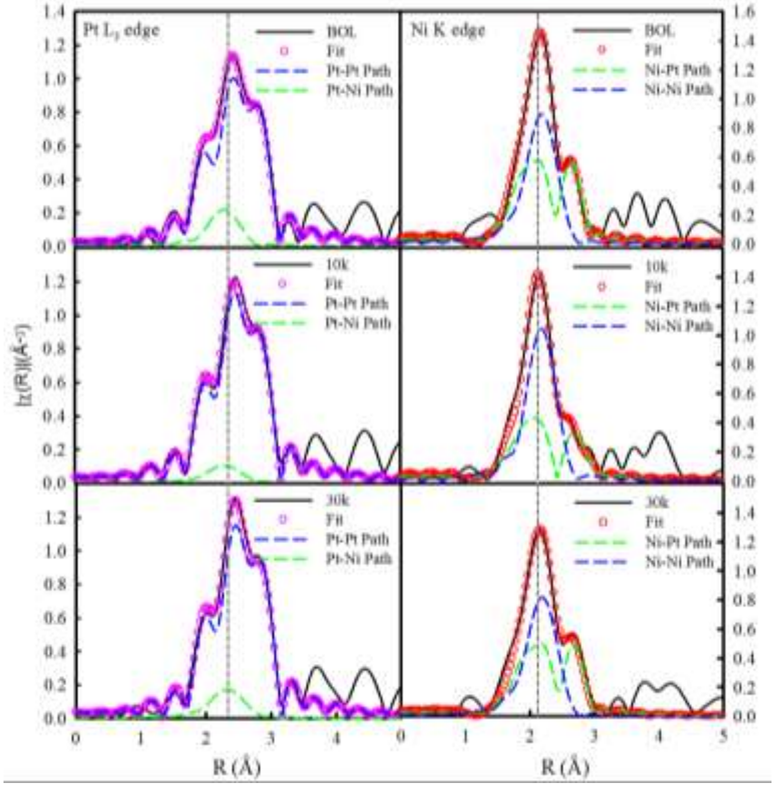

Figure S1. Pt L_3 edge (left) and Ni K edge (right) EXAFS spectra collected at 0.54 V in N_2-saturated 0.1 M $HClO_4$ electrolyte and the corresponding least-squares fits for dealloyed $PtNi_3/C$ (ANAu) NP catalyst under different voltage cycling stages: BOL (top), 10k (middle), and 30k (bottom). The vertical black lines are drawn as guides to the eye.

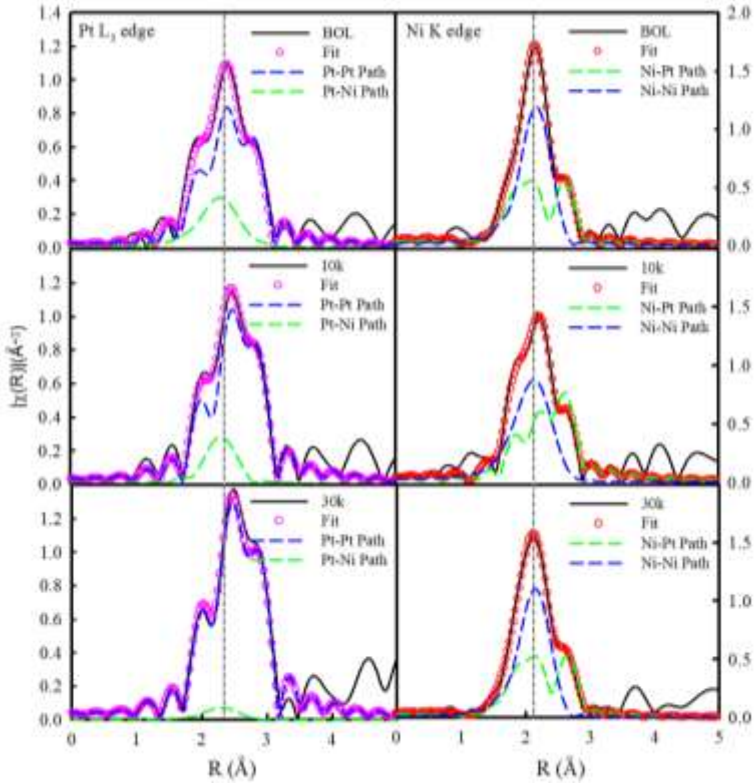

Figure S2. Pt L_3 edge (left) and Ni K edge (right) EXAFS spectra collected at 0.54 V in N_2-saturated 0.1 M $HClO_4$ electrolyte and the corresponding least-squares fits for dealloyed $PtNi_3/C$ (N_2NAu) NP catalyst under different voltage cycling stages: BOL (top), 10k (middle), and 30k (bottom). The vertical black lines are drawn as guides to the eye.

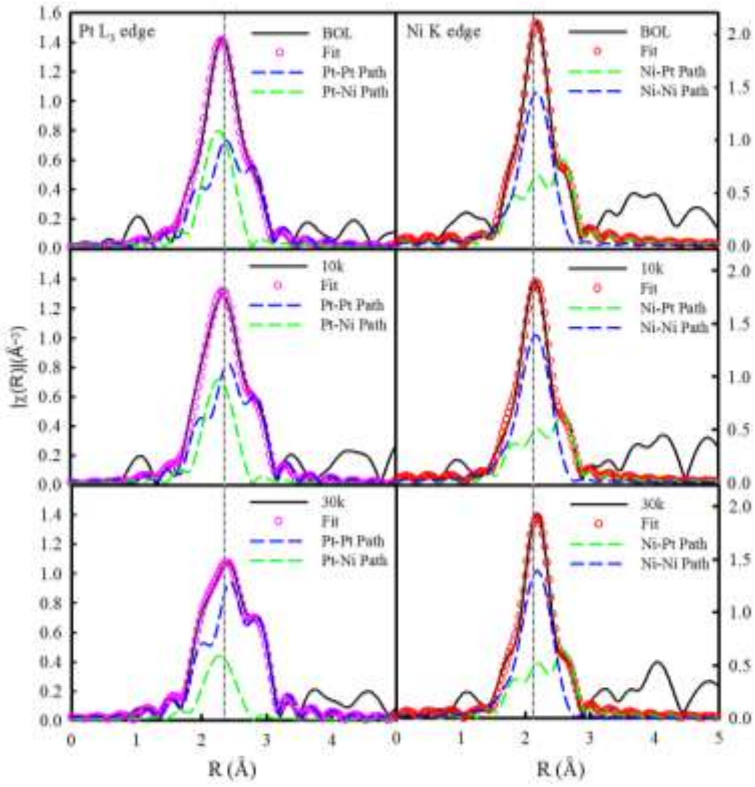

Figure S3. Pt L_3 edge (left) and Ni K edge (right) EXAFS spectra collected at 0.54 V in N_2-saturated 0.1 M $HClO_4$ electrolyte and the corresponding least-squares fits for dealloyed $PtNi_3$/C (N_2SAu) NP catalyst under different voltage cycling stages: BOL (top), 10k (middle), and 30k (bottom). The vertical black lines are drawn as guides to the eye.

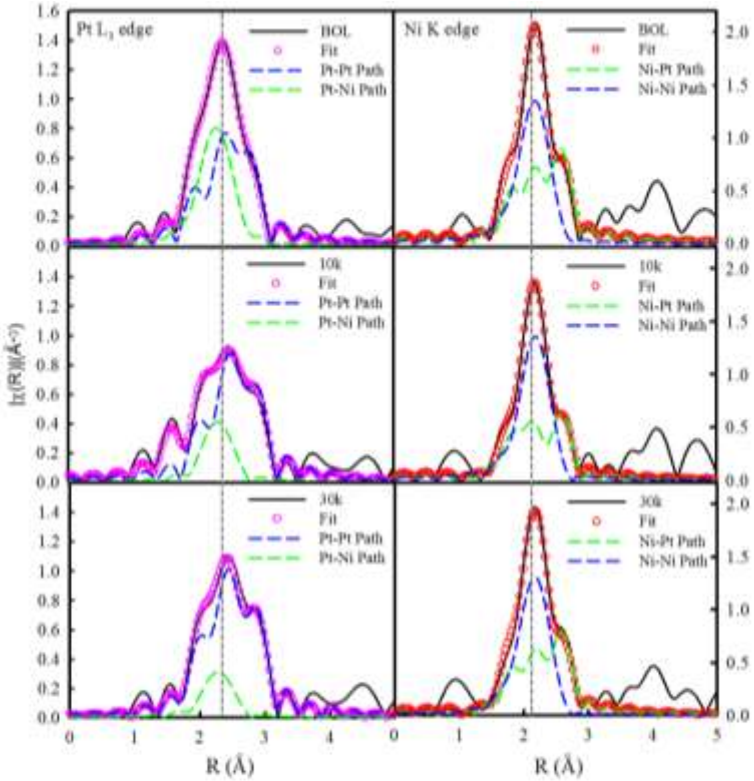

Figure S4. Pt L_3 edge (left) and Ni K edge (right) EXAFS spectra collected at 0.54 V in N_2-saturated 0.1 M $HClO_4$ electrolyte and the corresponding least-squares fits for dealloyed $PtNi_3$/C (N_2SAa) NP catalyst under different voltage cycling stages: BOL (top), 10k (the Pt L_3 edge data were collected under ex situ condition) (middle), and 30k (bottom). The vertical black lines are drawn as guides to the eye.

XANES data

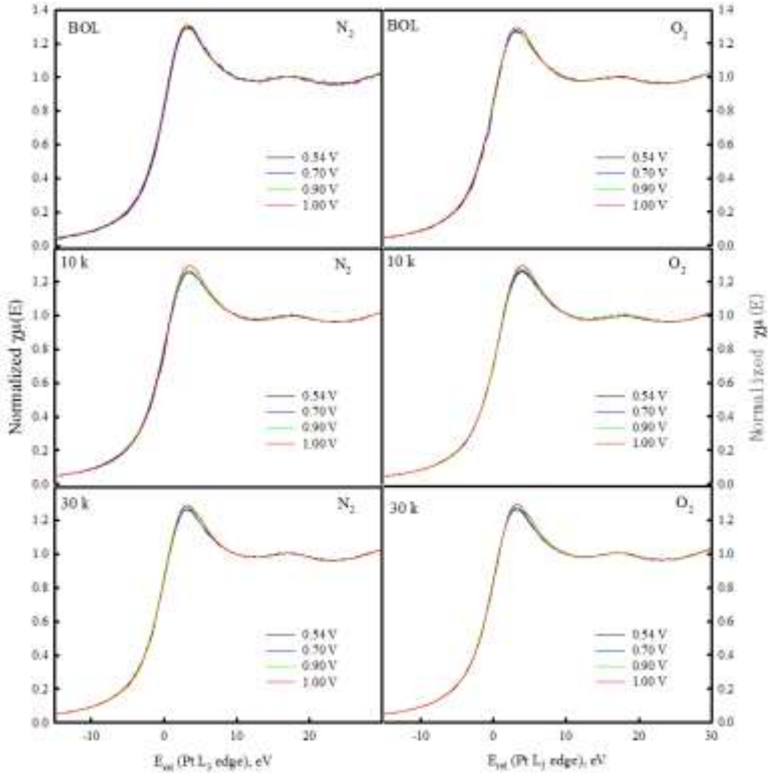

Figure S5. Normalized Pt L_3 edge XANES spectra collected at various potentials in N_2- (left) or O_2- (right) saturated 0.1 M $HClO_4$ electrolyte for dealloyed $PtNi_3/C$ (ANAu) NP catalyst under different voltage cycling stages: BOL (top), 10k (middle), and 30k (bottom).

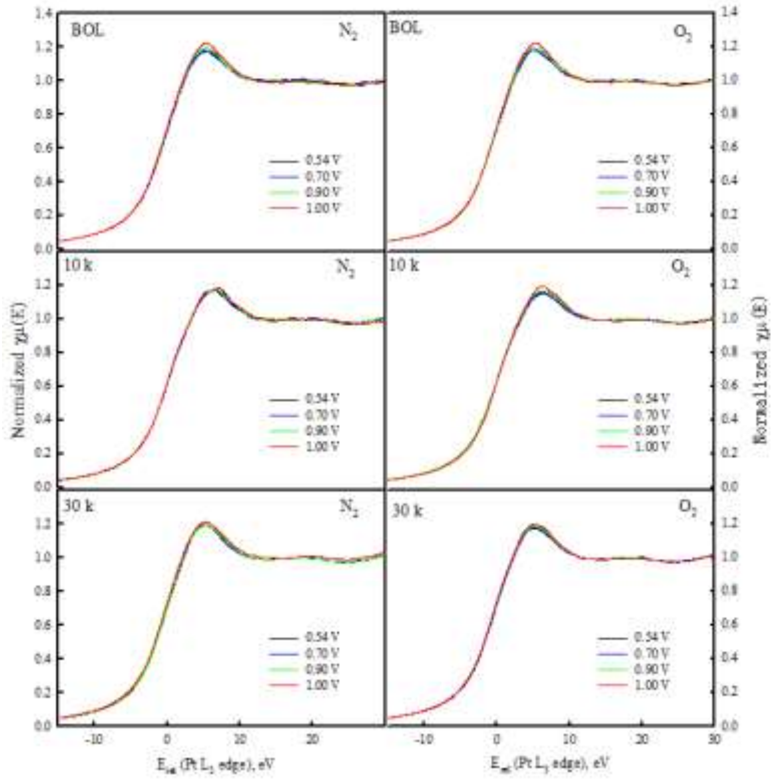

Figure S6. Normalized Pt L_3 edge XANES spectra collected at various potentials in N_2- (left) or O_2- (right) saturated 0.1 M $HClO_4$ electrolyte for dealloyed $PtNi_3/C$ (N_2NAu) NP catalyst under different voltage cycling stages: BOL (top), 10k (middle), and 30k (bottom).

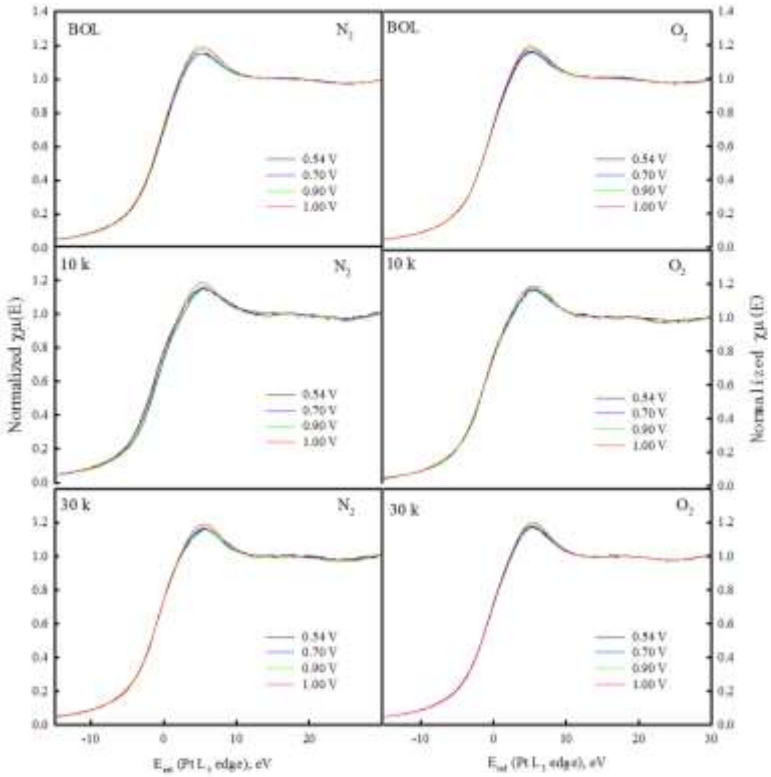

Figure S7. Normalized Pt L_3 edge XANES spectra collected at various potentials in N_2- (left) or O_2- (right) saturated 0.1 M $HClO_4$ electrolyte for dealloyed $PtNi_3/C$ (N_2SAu) NP catalyst under different voltage cycling stages: BOL (top), 10k (middle), and 30k (bottom).

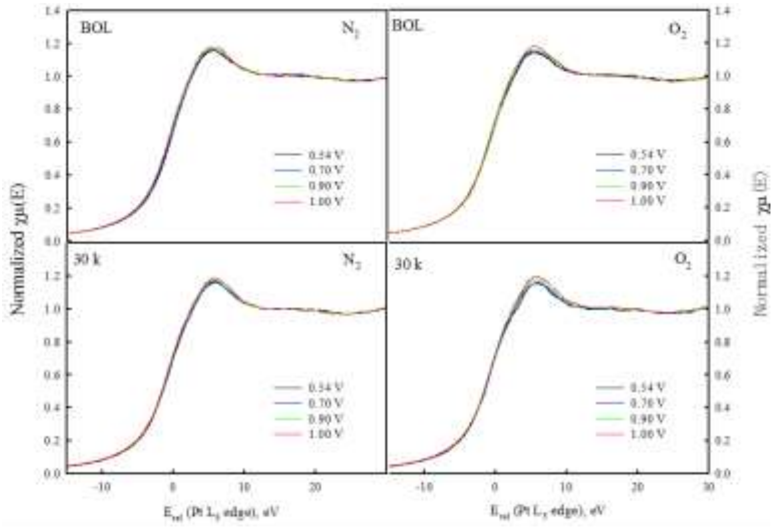

Figure S8. Normalized Pt L_3 edge XANES spectra collected at various potentials in N_2- (left) or O_2- (right) saturated 0.1 M $HClO_4$ electrolyte for dealloyed $PtNi_3$/C (N_2SAa) NP catalyst under different voltage cycling stages: BOL (top) and 30k (bottom).
